# Evidence for an enhanced HIV/AIDS policy and care in Cameroon: proceedings of the second Cameroon HIV Research Forum (CAM-HERO) 2021

**DOI:** 10.11604/pamj.2022.43.92.37080

**Published:** 2022-10-20

**Authors:** Patrice Tchendjou, Peter Vanes Ebasone, Anastase Dzudie, Eveline Mboh Khan, Joseph Fokam, Pius Tih Muffih, Alexis Ndjolo, Leonard Bonono Nyoto, Charles Kouanfack, Gabriel Mabou, Tatiana Djikeussi, Colette Sih, Jerome Ateudjieu, Boris Tchounga, Boris Youngui Tchakounte, Simplice Lekeumo, Felicite Naah Tabala, Benjamin Atanga, Leonie Simo, Madeleine Bakari, Armel Zemsi, Emile Shu Nforbih, Gilles Ndayisaba, Saint Just Petnga, Julie Laure Nguemo, Marc Lionel Ngamani, Phyllis Fon, Judith Nasah, Esther Neba, George Njie, Nicoline Ndiforkwah, Ezechiel Ngoufack Semengue, Tshimwanga Katayi, Gilbert Tene, Pascal Atanga Nji, Emmanuelle Njankou, Nyenty Agbornkwai, Appolinaire Tiam, John Ditekemena, Clement Ndongmo, Therese Abong Bwemba, Serge Clotaire Billong, Anne Cecile Zoung-Kany Bisseck, Louis Richard Njock

**Affiliations:** 1 Elizabeth Glaser Pediatric AIDS Foundation (EGPAF), Yaoundé, Cameroon,; 2 Clinical Research Education, Networking and Consultancy, Yaoundé, Cameroon,; 3 Faculty of Medicine and Biomedical Sciences, University of Yaoundé I, Yaoundé, Cameroon,; 4 Department of Internal Medicine and Subspecialities, Douala General Hospital, Douala, Cameroon,; 5 Lown Scholars Program, Department of Global Health and Population, Harvard T. H. Chan School of Public Health, Boston, USA,; 6 Cameroon Baptist Convention Health Services (CBCHS), Bamenda, Cameroon,; 7 Chantal Biya International Reference Centre for Research on HIV/AIDS Prevention and Management (CIRCB), Yaoundé, Cameroon,; 8 Faculty of Health Sciences, University of Buea, Buea, Cameroon,; 9 National AIDS Control Committee, Ministry of Public Health, Yaoundé, Cameroon,; 10 HIV Day Hospital, Yaoundé Central Hospital, Yaoundé, Cameroon,; 11 Faculty of Medicine and Pharmaceutical Sciences, University of Dschang, Dschang, Cameroon,; 12 Division of Health Operational Research, Ministry of Public Health, Yaoundé, Cameroon,; 13 Limbe Regional Hospital, Limbe, Cameroon,; 14 Bamenda Regional Hospital, Bamenda, Cameroon,; 15 Jamot Hospital, Yaoundé, Cameroon,; 16 Pan African Medical Journal, Yaoundé, Cameroon,; 17 Division of Global HIV and TB, Center for Global health, U.S, Center for Diseases Control and Prevention, Yaoundé, Cameroon,; 18 National Ethics Committee, Yaoundé, Cameroon,; 19 General Secretariat, Ministry of Public Health, Yaoundé, Cameroon

**Keywords:** HIV, Research, Cameroon, meeting report

## Abstract

To attain the HIV 95-95-95 goals by 2030 in Cameroon, high quality research to inform policy and patient care is of utmost importance. In the context of limited workforce and resources, collaborations, sharing of locally-adapted strategies and other field experience, leveraging on existing and innovative platforms would facilitate a coordinated and optimal AIDS response at country level. The second edition of the Cameroon HIV Research Forum (CAM-HERO) conference took place both physically and virtually on November 18 and 19, 2021 in Kribi, on the theme “Research for Policy and Care”. This scientific event brought together Cameroonian HIV/AIDS researchers, experienced clinicians and regulatory authorities to foster i) the dissemination of research findings and facilitate translation into policy, ii) operational research collaboration, iii) identification of new research areas, and iv) capacity building. To achieve the set objectives during this event, a consensus on research priorities for accelerating the achievement of three 95 HIV goals in Cameroon were summarized; meeting sessions included 31 abstract presentations, 13 discussions, and presentations on various aspects of HIV research including ethics, administrative procedures and needs for capacity building; training of young scientists on guidelines for research proposal development toward ethical clearance was done; and a platform for discussion between researchers and regulatory authorities was conducted around the design and setting-up of a national HIV/AIDS research agenda. CAM-HERO 2021 brought together HIV researchers, experts and junior scientists around major programmatic challenges, evidence to translate into practice, research priorities on HIV/AIDS. Collaborations were reinforced, capacities were strengthened, and footprints were established towards a consensus on a national HIV/AIDS research agenda.

## Conference Proceedings

### Introduction

To inform high quality policy making and patient care in HIV/AIDS, context specific research is essential. It is therefore imperative to bring together actors in the domain of HIV/AIDS in Cameroon towards the single goal of accelerating the attainment of the UNAIDS 95-95-95 goals in Cameroon. In 2020, these actors came together to form the Cameroon HIV/AIDS Operational Research Forum (CAM-HERO) [1]. CAM-HERO aims to improve and optimize the pertinence, performance, and impact on decision making of HIV research in Cameroon. More specifically CAM-HERO will help empower 1) Dissemination of research findings and facilitate translation into policy; 2) Operational research collaboration by facilitating collaboration among research organizations; 3) Identification of new research questions by improving collaboration with the Ministry of Health in the identification and answering of new HIV research priority questions; and 4) Capacity building. While the first meeting of CAM-HERO took place on the 10^th^ and 11^th^ of December 2020 at Hôtel Résidence Jully, Kribi and focused on issues related to ethics and HIV research, impact of COVID-19 on ART programs, Cameroon´s HIV research priorities, and opportunities for HIV research collaboration, the second CAM-HERO conference was held in Kribi on the 18^th^ and 19^th^ November 2021. The theme of this conference was “Research for Policy and Care”. At the 2021 event, the research priorities for accelerating the achievement of three UNAIDS 95 HIV goals in Cameroon were unveiled. Out of the more than 80 abstracts received, 16 were selected for oral presentation, 12 for poster presentations and 3 as late breaking abstracts. There was a wide range of discussions, ranging from ethics, administrative procedures, capacity building to ways to enhance effective collaboration. Of special note was the discussion on the way forward on the definition of a national HIV/AIDS research agenda.

### Day 1: November 18^th^, 2021

Immediately after Dr. Patrice Tchendjou, introduced and welcomed participants, the event was officially kick-started by Prof Anne Bissek, the Director of the Division of Health Operations Research (DROS) of the Ministry of Public Health. Prof. Bissek lauded the initiative and expressed her full support. Dr Serge Billong of the National AIDS Control Committee (NAAC) also appreciated the conference mentioning that it represents a huge opportunity for Cameroon to be a leader in Central Africa in advancing HIV research. The two-day event commenced with abstract sessions. These sessions included abstracts in the domains of preventive, clinical, implementation and basic sciences. The abstract sessions were chaired and moderated by panels of experts who assessed a broad range of interesting and insightful studies on HIV/AIDS science in Cameroon. Oral abstract presentation made during CAM-HERO 2021 were:

### Abstract session 1: prevention science

The first session of oral abstract presentations had as panel members Prof Anastase Dzudie Clinical Research Education Network and Consultancy - International epidemiologic Databases to Evaluate AIDS (CRENC-IeDEA), Dr. Patrice Tchendjou, Elizabeth Glaser Pediatric AIDS Foundation (EGPAF) and Dr Mboh K. Evelyne, Cameroon Baptist Convention Health Services (CBCHS). The following abstracts were presented. “Prevalence and Determinants of Reduced Glomerular Filtration Rate in HIV-Infected Patients on Antiretroviral Therapy at Bafoussam Regional Hospital” in which they found a reduction in glomerular filtration rate for antiretroviral treatment (ART) patients on Tenoforvir, Lamivudine, Effevarenz (TLE), by Paul Nyibio Ntsekendio. “HIV-Self Testing: A Strategy to Optimize Case Identification in Hard-To-Reach Populations” which had promising results but pointed to a need for close follow-up of the cases identified to ensure that they receive confirmatory tests and get enrolled on ART, by Banlack Ernest. “HIV Prevalence and Disease Outcome among Patient with Childhood Cancer in the Cameroon Baptist Convention Health Services” by Bernard Wirndzem Njodzeka. This study found that the children with childhood cancer were 4.5 times more likely to be HIV positive compared to children in the general population (prevalence of 1.8% compared to 0.04%), pointing to the need to take them to hospitals early. “Engaging Traditional Healers in HIV Testing Service Uptake and Care Cascade: A Randomized Controlled Trial in the South West Region of Cameroon” which led to increased knowledge and practices such as counselling and referral of positive cases to hospitals for additional care, by Charlotte Ayima.

### Abstract session 2: clinical science

Panel members for this segment of oral abstract presentations were Prof. John Ditekemena (EGPAF), Dr. Pascal N. Atanga, (CBCHS) and Dr. Joseph Fokam, Centre International de Référence Chantal BIYA, Faculty of Health Sciences - University of Buea (CIRCB, FHS-UB). The presentations included:

“Association between Cervical Neoplasia and CD4 Counts among Women Living with HIV in Cameroon” presented by Manjuh Florence. The study found strong evidence of an association between a positive cervical cancer screening outcome and low CD4 counts (OR= 1.44; P= 0.0004), highlighting the importance of screening all women on ART for cervical cancer and call for the integration of cervical cancer screening into HIV programs, which could greatly reduce morbidity and mortality in females.

“Service Provision during weekends and extended clinic hours: an effective differentiated patient-centered approach for HIV Care and Management” presented by Ismaila Esa. This retrospective study conducted in 25 CDC/PEPFAR supported facilities during a 9-month period showed that 7,666 clients were tested for HIV with 434 new positives identified, linked and started on ART; a total of 1,226 clients on ART came to access services, and 2,355 had their samples collected and sent to the reference laboratory for viral load testing. Hence service provision during weekends and extended clinic hours has the potential to significantly improve the uptake of clinical services in HIV programs.

“Treatment Outcomes and Factors Associated to Treatment Attrition 12 Months After ART Initiation in A Large Cohort of HIV Positive Clients in The West Region of Cameroon” presented by Tshimwanga Katayi Edouard. In this study, of a cohort of 4,097 HIV+ identified, 3,799 (92.6%) initiated ART. After 12 months of follow-up, 1,955 (84.3%) of clients were still on treatment, 147 (6.3%) transferred out, 97 (4.2%) were lost to follow-up, 97 (4.2%) died, 23 (1.0%) stopped treatment. The factors associated to attrition (Dead, LTFU and Stop ART) were male gender (AOR= 1.6, p<0.001) and WHO Clinical Stages 3&4 (AOR= 4.0, p<0.001; AOR= 12.7, p<0.001).

“Personalized HIV Medicine Improves Antiretroviral Treatment Outcomes Among Adolescents in Cameroon: Experience from the EDCTP Ready-Study” presented by Willy Leroi Togna Pabo. Here, the authors sought to evaluate the effect of HIV-1 mutational profiling on immuno-virological response and acquired drug resistance (ADR) among adolescents living with perinatal HIV. Predictors of ADR were being on first-line ART (p=0.045), virologic failure at baseline (OR=12.56, 95%CI 2.32-68.13, p=0.059), and immunological failure (OR=5.86, 95%CI 1.18-29.04, p=0.010). Inversely, good adherence (OR=0.13, 95%CI 0.02-1.10, p=0.0003), and optimised ART following mutational-profile (OR=0.05, 95%CI 0.01-0.41, p=0.002) were protective factors.

### Abstract session 3: implementation Science

Panelists for this session were Prof. Anne Bissek Division de la Recherche Opérationnelle en Santé /Ministere de la Sante Publique (DROS/ MSP), Dr Charles Kouanfack, Hopital Central de Yaounde, Hopital Central de Yaounde (HCY, UDS) and Dr Serge Billong, National AIDS Control Committee (NACC). Abstracts included: Abstracts included: “Differentiated HIV Testing Models and Case Identification in Three Regions of Cameroon” by Tshimwanga Edouard. Here, three testing modalities were implemented from 80 health facilities. The modalities were 1) Provider Initiated Testing and Counseling at health facilities, 2) Index Testing, and 3) Health facility-Led targeted Community Testing which respectively led to 3.7%, 9.9%, 1.4% yield, and their contribution to positives were 50% 26%, and 24%.

“Enhanced adherence counselling, support groups and viral load suppression amongst adolescents at Centre Hospitalier d´Essos” by Agbornkwai Agbor. This study showed that Suppression rates were good after the completion of EAC sessions and enrolment and participation in support groups for adolescents with a high viral load.

“Establishing Enhanced Adherence Counselling (EAC) to Monitor and Appropriately Manage High Viral Load (HVL) ART Children/Adolescents at The CBCHS Supported Sites in the West, Northwest and Southwest Regions of Cameroon” by Gilbert Tene. Here the study showed that EAC helped differentiate HVL from poor adherence needing adherence support, and HVL from true ART failure needing switching.

“Is there an Association Between the Home-Based Care Provider for Children and Adolescents on Antiretroviral Therapy (ART) and Their Viral Suppression Status? Evidence from Cameroon” by Eveline Mboh. Findings from this study suggest that home-based care provider has no relationship with viral suppression.

### Abstract session 4: basic science

The fourth session’s panelists were, Prof. Pius Tih (CBCHS), Prof. Jerome Ateudjeu, Faculte de Medecine et des Sciences Pharmaceutiques, Universite de Dschang (FMPS, UDS), and Dr. Tene Gilbert (CBCHS). The abstract presentations included:

“Predictors of immune response adolescents with perinatal HIV infection in Cameroon” by Aurelie Minelle Kengni Ngueko. This communication showed the challenges in achieving a good treatment response among adolescents with poor immunity, and raised in need to continuously provide CD4-monitoring for an enhanced clinical management of children/adolescents with advanced disease in the context of HIV infection in Cameroon.

“Effect of HIV-1 Genetic Diversity on Immune-Virologic Response Among Adolescents in Cameroon: Experience from The EDCTP Ready-Study” by Willy Leroi Togna Pabo. This communication revealed the high burden of virological failure and the poor immunity of vertically-infected adolescents receiving ART in Cameroon, the braod HIV-1 diversity as well as the similar treatment outcomes irrespective of viral subtypes and recombinants.

“Incidence and Factors Associated with Virologic Failure in Adult People Living with HIV (PLHIV) with Previous Viral Load Suppression at the Jamot Hospital Yaoundé” presented online by Tentoum Claire Aimée. In this study, the authors reported that one out of 14 adult PLHIV enrolled in care experienced virologic failure over 24 months after achieving viral load suppression. Patients with a baseline CD4 count less than 100 cells/mm^3^ were at higher risk of virologic failure after initial viral suppression; and should therefore benefit from more rigorous follow up.

“Pre-Treatment HIV Drug Resistance in Cameroon and Implications on First-Line Therapeutic Options” by Collins Chenwi. This communication showed the patterns of antiretroviral drug resistance among newly diagnosed patients and the adequacy with dolutegravir-based first-line regimens, which indicated a very high potency and genetic barrier to resistance within the context of ART program in Cameroon. These oral abstract sessions were very insightful and well conducted. The panel members made remarkable critiques of the presentations and evoked strong take away messages from the presentations. The general audience was equally given the opportunity to ask questions or contribute.

### Plenary session I: opportunities for effective collaboration between researchers, ethics committees and regulatory bodies

This session had two discussion points:

*Discussion 1: from submission to ethical clearance in Cameroon: what are the challenges and solution?*

This first discussion of this plenary session began with talks by Dr. Boris Tchounga and Dr. Mboh K. Eveline who shared their experiences with the process of obtaining ethical clearance. This was followed by a presentation by Dr. Thérèse Abong, the president of the National Ethics Committee. The presentation was titled “*Ethical review of research in Cameroon: Current processes and strategies*”. At the end of these presentations, the discussants recommended the following i) setting up a website to communicate procedures for ethical submission and review of research protocols, ii) increasing committee members to increase frequency of sessions the ethical committee panel meets to review research protocols, iii) a proposal to set up a permanent secretariat that can do expediated reviews for low-risk studies.

*Discussion 2: research Regulation & HIV policy*

The session comprised of two main topics. The first part of the session was a presentation on “*Administrative authorization for research: tips for the busy researcher*” by Mme Naah Felicité of DROS and the second was “*Ethics committees in Cameroon: Organization of Regional and Institutional Committees: what researchers need to know*” by Prof. Jerome Ateudjeu. These presentations were accompanied by enthusiastic interactions between the session chairs, presenters and the audience. The key points arising from these discussions were i) researchers should ensure to get administrative approval prior to starting study implementation, ii) there is a need to ameliorate the process and time for obtaining ethical clearance from the national ethics committee and iii) to accelerate the opening of regional ethics committees. The first plenary session was closed by Dr. Joseph Fokam who made an insightful presentation on “HIV mutations in the era of COVID-19 epidemic: myths or facts”. Basic concepts on HIV genetic variability were elucidated, interactions between HIV and COVID-19 was unveiled; experience and challenges in managing people living with HIV during the COVID-19 epidemic in Cameroon were shared, and major knowledge gaps in HIV/COVID-19 coinfection were addressed.

### Plenary session II: late breaking abstracts

Important, breaking and high-quality abstracts were reserved for this special segment of the event. The three late breaking abstracts were:

“Challenges in the Management of HIV Patients at an Advanced Stage of the Disease. Preliminary Results of the Driving Reduced AIDS-associated Meningo-encephalitis Mortality (DREAMM) study was presented by Dr Charles Kouanfack." The study shows that significant number of deaths of critically unwell PLHIV are preventable using existing diagnostic and treatments; and that Toxoplasmosis is the most prevalent infection of the Central Nervous System in Cameroon.

“New Horizons Project (advancing pediatric HIV care): Pattern of Baseline HIV-DR Among and Outcomes of Patients receiving Etravirine/Darunavir at 6-12-month follow-up” by Dr Appolinaire Tiam. The study found that Highly treatment-experienced children/adolescents in sub-Saharan Africa have accumulated high-level of resistance to NRTI, NNRTI, and commonly used PIs, but susceptibility to darunavir (DRV) and second generation NNRTI was retained in most cases.

“COVID-19 associated changes in HIV service delivery over time in Central Africa: Results from facility surveys during the first and second waves of the pandemic” presented online by Dr Rogers Ajeh. Here, the authors showed that while the initial wave of the COVID-19 pandemic resulted in disruptions to HIV service delivery, most of these disruptions appear to have attenuated over time, and many sites introduced measures to help people living with HIV avoid frequent visits to the clinic for care and medications.

### Day 2: November 19^th^, 2021

After a recap of day 1 was read and adopted, the meeting started with the third plenary session.

### Plenary session III: research methodology

This session included two presentations. The first on “Priority research areas to accelerate the 95s in Cameroon” by Prof. Anastase Dzudie. Prof Dzudie presented the 5 HIV research priorities determined during the 1^st^ CAM-HERO conference in 2020 and published in the Pan African Medical Journal [2]. He reported that, those priorities could be redefined every five years and suggested that CAM-HERO needs to engage researchers to make use of these identified priority areas. The second presentation for this plenary session was given by Prof. John Ditekemena on “Principles of implementation research”. Prof Ditekemena´s talk was very rich and practical. The aim of his presentation was to emphasize on how to develop and implement evaluation protocols in program activities; as well as other research specific protocols (cohort study; trial; case control study). In addition, during this presentation, participants were taught on how to build a strong research/ Evaluation team as well as how to develop ethics and good practice in research. Both presentations were appreciated by the audience and the outcomes were applauded.

**Workshops: collaborative research & research registry:** the round table workshop sessions were on two key aspects.

### Fostering collaborative research in Cameroon

This session was chaired by Prof. John Ditekemena and Dr Thérèse Abong and had two presentations. Dr Appolinaire Tiam gave the first talk on “*How to design a good research question*”. Key points arising from this talk were the need to strengthen mentor-mentee relationships, enhance capacity building, mapping of funding sources and including biostatisticians in subsequent CAM-HERO training sessions. Prof Anastase Dzudie then took the floor to present on “*Ways to increase collaboration in Cameroon*”. His talk highlighted the need to enhance a win-win collaboration within and outside the country by contextualizing research activities to fit our setting, promote capacity building within projects in the country and mapping and linking researchers under the leadership of the DROS. Also, identifying funding sources and teaming up in grant applications.

### Developing and launching a Cameroon HIV/AIDS research registry (CamnARR)

The second workshop was chaired by Prof Anne Bissek and Dr Serge Billong. Prof. Anastase Dzudie made a presentation proposing the creation of a Cameroon National HIV/AIDS Research Registry Platform (CamNARR) that will serve as a database of studies on HIV/AIDS research in Cameroon. This will help in aggregating studies in one place for easy access and prevent duplication of efforts. The idea was highly received, and some remarkable contributions were made. At the end of the workshop sessions, a representative of The Pan African Medical Journal (PAMJ) then took the floor to introduce the journal and its services. Discussions were equally held on how PAMJ could be an asset and collaborator necessary for ensuring the CAM-HERO vision.

### CAM-HERO Awards

**Figure 1:**
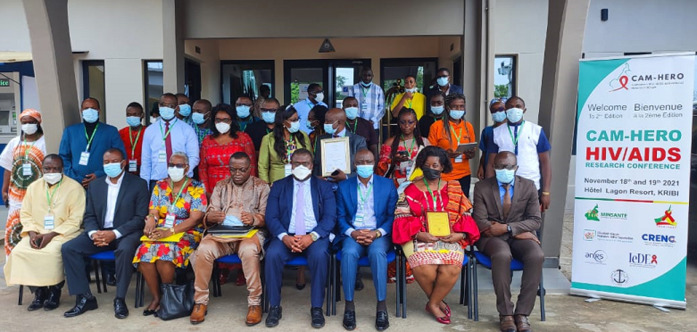
group photo during the 2021 edition of the Cameroon HIV Research Forum

The last part of the two-day event was the CAM-HERO Awards. It was presided over by the Secretary General of the Ministry of Public Health, Prof. Richard Njock. Prof. Njock made an astonishing speech in which he congratulated the CAM-HERO organizers and attendees for their dedication to advancing HIV/AIDS research in Cameroon. A handful of prominent researchers were awarded certificates of achievement in recognition of their tireless dedication to HIV/AIDS research in Cameroon. The first best oral abstract presentation was awarded to Collins Chenwi, the second-best oral abstract to Dr Gilbert Tene and best poster award went to Chiabi Eugene. Special awards of excellence in leadership were offered to Prof. Richard Njock, Prof. Anne Bissek and Dr. Thérèse Abong. The team took family pictures (Figure 1) and the meeting ended.

### Funding

The Kribi meeting was funded by the Cameroon research group of the Elizabeth Glaser Pediatric AIDS Foundation (EGPAF), the Cameroon Baptist Convention Health Services (CBCHS), and the International epidemiology Databases to Evaluate AIDS-Clinical Research Education Networking and Consultancy research group (CRENC-IeDEA).

**Disclaimer:** the findings and conclusions in this report are those of the authors and do not necessarily represent the official position of the agencies. No specific funding was used for the development of the paper.

## Conclusion

The second edition of CAM-HERO brought together key stakeholders together in Kribi to share new research findings in the country, discuss research ethics and administrative procedures, research methodology, research priority areas, ways of fostering collaboration and innovations to make HIV/AIDS research more accessible to all in Cameroon. It was a fruitful event marked by the publication of the national HIV/AIDS research priorities and discussion to pave of the way for a national HIV/AIDS research agenda, which are critical components for achieving the UNAIDS 95-95-95 goals in Cameroon.
